# Preferences of women with epithelial ovarian cancer for aspects of genetic testing

**DOI:** 10.1186/s40661-019-0066-8

**Published:** 2019-01-22

**Authors:** Brittany A. Davidson, Jessie Ehrisman, Shelby D. Reed, Jui-Chen Yang, Adam Buchanan, Laura J. Havrilesky

**Affiliations:** 1 Department of Obstetrics and Gynecology, Division of Gynecologic Oncology, Duke University Medical Center, Duke Cancer Institute, Box 3079, Durham, NC 27710 USA; 2 Duke Clinical Research Institute, Duke University, Durham, USA; 3 Department of Population Health Sciences, Duke University, Durham, USA; 4 Geisinger Genomic Medicine Institute, Danville, PA USA

**Keywords:** Ovarian cancer, Genetic testing, Patient preferences

## Abstract

**Background:**

Although genetic testing is recommended for women with epithelial ovarian cancer (EOC), little is known about patient preferences for various testing options. We measured relative preferences for attributes of testing in women with EOC referred for genetic counseling.

**Methods:**

Subjects were recruited to participate in a discrete-choice-experiment survey to elicit preferences for attributes of genetic testing: out-of-pocket cost ($0, $100, $250, or $1000), probability of a deleterious mutation (60, 80%, or 88%), probability of a variant of uncertain significance (VUS) result (5, 20%, or 40%), sample requirements (blood or saliva), and turn-around time (1, 2 or 4 weeks). Subjects viewed educational videos followed by a series of choices between pairs of constructed genetic tests with varying attribute levels. Random-parameters logit was used to estimate preference weights for attribute levels. Relative importance weights and money-equivalent values were calculated.

**Results:**

Ninety-four patients were enrolled; 68 (76.4%) presented for genetic counseling. Test cost was the most important attribute to subjects (importance weight = 41 out of 100) followed by probability to detect deleterious mutations (36) and probability of a VUS result (20). Sample requirements and turnaround time did not drive test choices. Subjects were willing to pay an additional $155 and $70 for incremental 5% improvements in the probability to detect deleterious mutations and probability of a VUS result. At genetics consultation, 55/68 (80.9%) subjects chose multigene testing.

**Conclusions:**

Low out-of-pocket cost, high probability of detecting deleterious mutations and high probability of a VUS result are preferred by patients with EOC considering genetic testing.

**Electronic supplementary material:**

The online version of this article (10.1186/s40661-019-0066-8) contains supplementary material, which is available to authorized users.

## Introduction

Personalized medicine is transforming contemporary cancer care. Germline genetic testing is an expanding and increasingly visible means to modify personal cancer risk. However, the evidence base surrounding how patients with cancer value various features of genetic testing is under-developed. Nearly one-quarter of epithelial ovarian cancers (EOC) now have an identifiable hereditary cause [1]. Although the majority of hereditary EOC is associated with mutations in either *BRCA1* or *BRCA2* (65–85%) [2], genes in the homologous recombination and mismatch repair pathways have also been implicated in hereditary ovarian tumorigenesis [1, 3]. Hereditary EOCs are also associated with elevated risks of other malignancies, including breast, uterine, and colorectal cancers. As such, in March 2014 the Society of Gynecologic Oncology (SGO) issued guidelines stipulating that all women with a diagnosis of EOC should be offered genetic counseling, with consideration given to genetic testing [4].

The results of genetic testing may have a significant impact on the physical, emotional and financial well-being of those who are tested and their families. The identification of hereditary genetic defects in women with EOC may lead to directed gene testing in family members and enhanced screening recommendations or medical or surgical prophylaxis for associated cancers. Patients’ concerns about the potential financial difficulties associated with testing and downstream medical monitoring as well as concerns about insurance or employment discrimination may also impact their willingness to undergo genetic testing [5].

Meanwhile, a battery of new genetic tests is flooding the market, with no clear guidance for patients or clinicians regarding the appropriate order of testing. Testing for single genes that are part of well-recognized syndromes, such as the *BRCA1/2* genes, is most likely to provide a definitive result with actionable clinical care recommendations. However, a number of currently marketed genetic panels include up to 35 genes. Results from these panels may identify deleterious mutations in additional genes known to increase the risk of certain malignancies. Variants of uncertain significance (VUS), whose clinical relevance is unknown, may also be identified during testing. As a result, up to 40% of women who are tested with the more comprehensive panels will be identified as carriers of VUS [6], for which no clear pathologic implications or clinical management strategies have been identified.

Given the introduction of numerous genetic tests with varying attributes, our study aimed to elicit the preferences of women with EOC referred for genetic counseling in order to tailor genetic counseling and test selection. We quantified tradeoffs among 5 attributes of genetic testing strategies that would be considered acceptable to patients. We were specifically interested in the influences of out-of-pocket costs, the likelihood of identifying a deleterious mutation, and the likelihood of identifying a VUS.

## Methods

### Subject recruitment

Approval was obtained from the Duke University Institutional Review Board. Eligible subjects were identified by their gynecologic oncologist and offered study participation prior to being approached by a dedicated study coordinator. Eligible participants included English-speaking women > 18 years old with a diagnosis of non-mucinous EOC referred for germline genetic testing. Women with a family or personal history of cancer were included. Exclusion criteria were women < 18 years of age, non-English speakers and those unable to provide informed consent.

### Survey instruments

#### Discrete choice experiment

Discrete-choice experiments (DCE) are a specific type of conjoint analysis that are used to quantify preferences for various attributes of medical care, health states or other services [7–9]. Subjects are asked to choose between alternative constructed profiles characterized by specific levels of each attribute. When repeated iterations of this exercise are performed, data are generated to statistically estimate rates of acceptance of tradeoffs among the included attributes. This study was conducted to ascertain the preferences for 5 specific attributes relevant to genetic testing options among women with EOC.

#### Educational video

Each subject viewed a 7-min educational video to familiarize her with genetic testing, the 5 key attributes being studied, and their associated levels (Table 1) (Genetic Testing Education Video, see Additional file 1 for video text). This was developed by a team of gynecologic oncologists and genetic counselors at our institution.

**Table 1:** Attributes of genetic tests

Attributes	Levels
Out-of-pocket cost	● $0 ● $200 ● $1000 ● $5000
Probability of detecting a deleterious mutation	● 88% ● 80% ● 60%
Probability of a VUS result	● 5% ● 20% ● 40%
Sample requirements	● Saliva/Cheek swab ● Blood
Turn-around time	● 1 week ● 2 weeks ● 4 weeks

*VUS* variant of uncertain significance

#### Selection of attributes and corresponding levels

Selection of attributes was performed based on a literature review and consultation with genetic testing counselors. At the time of study development, poly ADP ribose polymerase (PARP) inhibitors were not yet FDA approved; thus, potential therapeutic implications were not included as attributes. The levels for each attribute were chosen to represent ranges inclusive of those that might be encountered in the various current genetic testing strategies that were available topatients at the time of study design.

The five attributes of genetic testing included:

1. Out-of-pocket cost: Since many patients face significant cost-sharing with genetic testing, an attribute representing out-of-pocket costs was included as an attribute to evaluate the extent to which patients would pay money to gain improvements in desirable testing features. (Levels: $0, $200, $1000, $5000)
2. Probability of identifying a deleterious mutation: The concept of a deleterious mutation was introduced and the probability of detection of a mutation, if one exists, was described for both single gene (*BRCA1/2* only) and multi-gene testing. Single gene testing was described as testing for mutations in only the *BRCA 1* & *2* genes, the most common genes associated with hereditary breast and ovarian cancer. Information regarding less common genes would not be available as a result of single gene testing. Multi-gene testing was described as testing for up to 25 different genes, including *BRCA1* and *2*. Subjects were informed that mutations in any one of these genes may influence their risk for several cancers, including breast, ovarian, colon and uterine. The more genes that are tested, the more likely it is that testing will find a deleterious mutation. (Levels: 88, 80, 60%)
3. Probability of VUS result: VUS were defined as changes present in a gene but with an unclear influence, and possibly no influence, on an individual’s cancer risk. Subjects were informed that if genetic testing identified a VUS, their doctor would not be able to tell them anything new about their or their family’s cancer risk. It was further explained that updated information may become available in the future that may clarify a VUS’ specific cancer risk. (Levels: 5, 20, 40%)
4. Sample requirements: Specimen collection was presented as either a blood draw or saliva sample. (Levels: Saliva/cheek swab, blood)
5. Turn-around time: Turn-around time was defined as the time interval between specimen collection and availability of results. (Levels: 1 week, 2 weeks, 4 weeks)

#### Experimental design

**Fig. 1:**
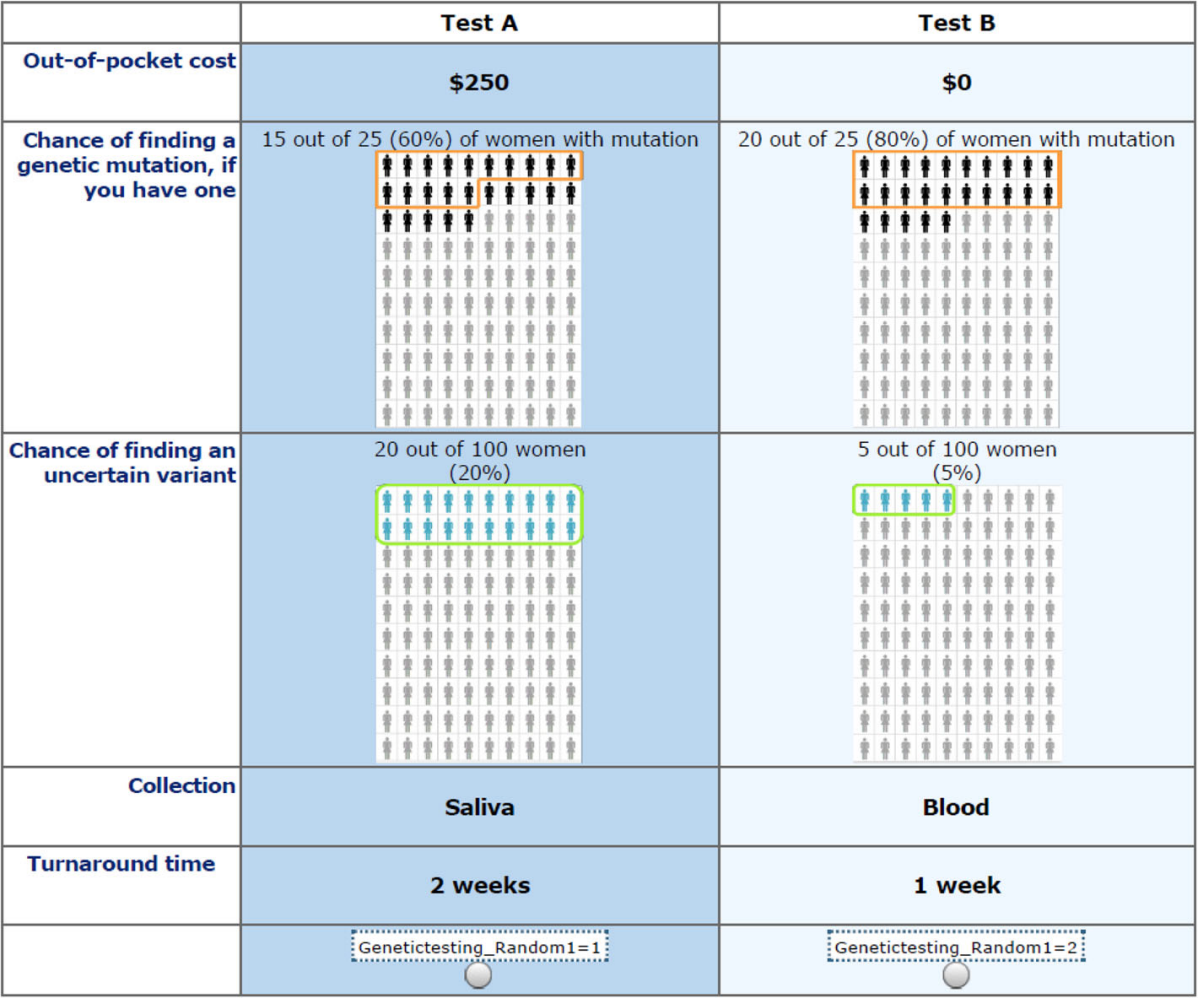
Example choice question

An experimental design consisting of 21 blocks of ten 2-alternative choice sets was created using the attributes and levels described above. Figure 1 is an example choice question from the experimental design.

#### Statistical analysis

We used random-parameters logit regression in Stata [10] to model subjects’ choices as a function of attribute levels and to obtain log-odds estimates representing relative preference weights for each attribute level. Specification tests suggested linear relationships for all numeric attributes except turn-around time. The sample requirements and turn-around time attributes were modeled using effect-coded variables. With effect coding, 0 indicates the mean effect across levels within an attribute such that parameter estimates for each level within an attribute is interpreted relative to the mean rather than the omitted level when interpreting parameter estimates using dummy coding. This procedure allows each attribute level to have an estimated preference weight. All preference weights were rescaled on a scale of 0 to 10, where 0 was assigned to the worst level (i.e., the smallest preference weight) of the important attribute and 10 was assigned to the best level (i.e., the largest preference weight) of the same attribute. Confidence intervals (CIs) around the preference weights were estimated using the Krinsky-Robb procedure with 10,000 draws and then rescaled accordingly [11]. For attributes with a linear specification, CIs were mean centered. If the CIs for different attribute levels do not overlap, the preference weights are statistically significantly different from each other at better than the .06%% level. Unobserved preference heterogeneity among subjects was modeled as a continuous distribution of preferences for each attribute level with the exception of out-of-pocket cost that was assumed to be a fixed parameter [12, 13].

To facilitate the interpretation of attribute relative importance, importance weights were calculated as a fraction of 100, with the weights for all attributes summing to 100. In addition, the parameter estimate for the cost attribute was used to calculate money-equivalent values (MEV) representing the additional amount of money that subjects would pay for tests with more preferred features. MEV is calculated as the utility provided by an increase in test precision (increasing test sensitivity for a deleterious mutation or probability of a VUS) scaled by the utility provided by $1.00 (the absolute value of the cost coefficient). CIs for the importance weights and MEVs were estimated using the Krinsky-Robb procedure with 10,000 draws [11]. Subsequent subgroup analyses examined the stated preferences for test attributes stratified by the method of genetic testing chosen by each subject for herself.

#### Pilot subjects

The first 4 subjects recruited to the study were used to pilot the survey instrument, educational video and DCE activities. Informal qualitative interviewing techniques were used to assess ease of use and level of understanding of the information provided.

## Results

One hundred-fourteen patients were consented. Of these, 20 subjects were excluded: 4 pilot subjects, 4 screen failures (met with genetics prior to survey completion), 1 duplicate, and 11 who did not complete the survey. A total of 94 subjects were included in the analysis. The majority of subjects enrolled had newly diagnosed EOC (74/94; 78.7%) and were on treatment (63/94; 67%). Most subjects were Caucasian (78/94; 83%) with a median age of 66 (range: 37–82). Serous histology was most common (74.5%), followed by adenocarcinoma NOS (8.5%), endometrioid (7.4%) and carcinosarcoma (1.1%). Nearly half (48.9%) of patients had a family history of breast and/or EOC.

**Fig. 2:**
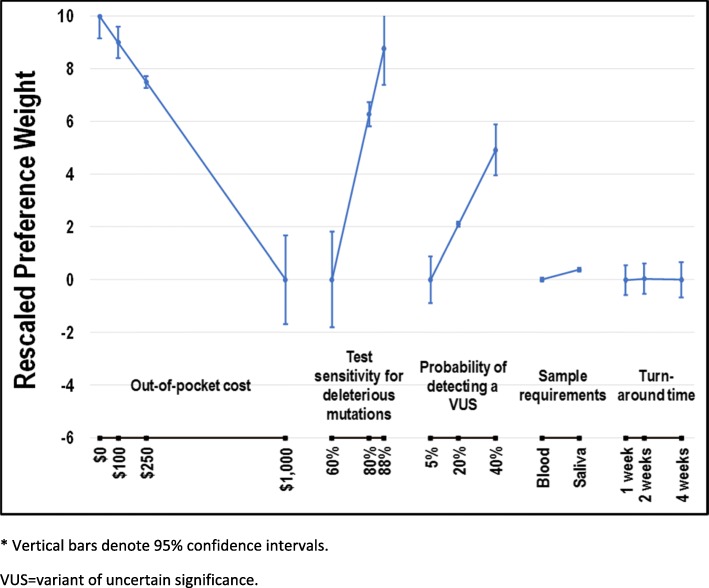
Rescaled preference weights* (*N* = 93). * Vertical bars denote 95% confidence intervals. VUS = variant of uncertain significance

Figure 2 displays preference weights scaled relative to test cost. In general, preference weights were consistent with the natural ordering of the levels; that is, better clinical outcomes were preferred to worse clinical outcomes. As expected, subjects preferred tests that provided better probability of identifying a deleterious mutation. On average, subjects would pay an additional $155 for a 5% incremental increase in detection from the baseline of 60%. Improved probability of VUS detection was also preferred. For a 5% increase in the probability of VUS detection from the baseline of 5%, subjects would pay an additional $70 out-of-pocket. MEVs of interest are reported in Table 2. Subjects also preferred test samples to be collected via saliva/cheek swabs over blood samples, although this difference was not statistically significant. Subjects showed no discernible preferences for any of the turnaround time levels.

**Table 2:** Money-equivalent values for select tradeoffs

Attribute	Improvement		MEV (95% CI)
	From	To	
Probability of detecting a deleterious mutation	60%	88%	$877 ($583 - $1226)
	80%	88%	$251 ($167 - $350)
	60%	80%	$627 ($416 - $875)
Probability of a VUS result	5%	40%	$492 ($305 - $718)
	20%	40%	$281 ($174 - $411)
	5%	20%	$211 ($131 - $308)

*CI* confidence interval, *MEV* money-equivalent value, *VUS* variant of uncertain significance

**Fig. 3:**
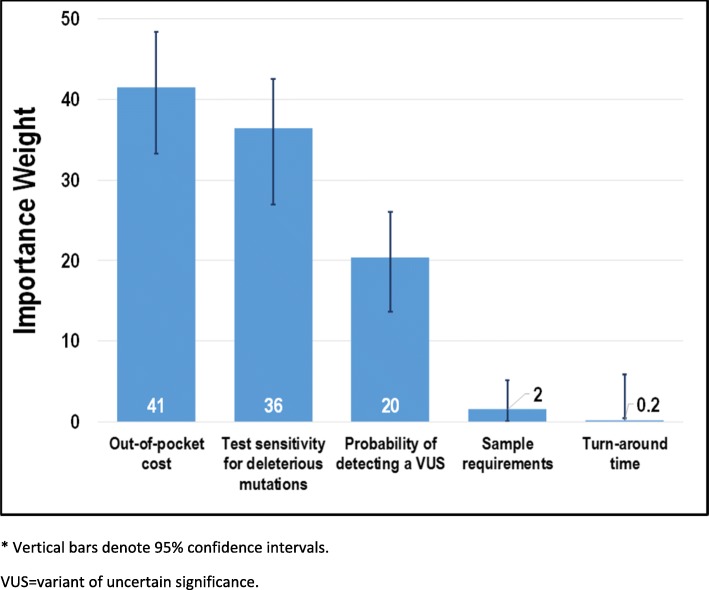
Importance weights* (*N* = 93). * Vertical bars denote 95% confidence intervals. VUS = variant of uncertain significance

Figure 3 displays the importance weights for test attributes. Among the 5 attributes evaluated in the study, test cost was the most important attribute to subjects with an importance weight of 41 (95% CI: 33–48), followed by the probability to detect a deleterious mutation (36 [27–43]) and VUS (20 [13–25]). Sample requirements (2 [0.09–5]) and turnaround time (0.2 [− 0.08–5]) were the least important attributes.

Of 94 participants, 69 (73.4%) subsequently chose to attend a genetic counselling appointment. At subsequent genetics consultations, 55/69 (79.7%) subjects chose multigene testing, 8/69 (11.6%) chose *BRCA1/2* testing only and 6/69 (8.7%) declined testing.

**Fig. 4:**
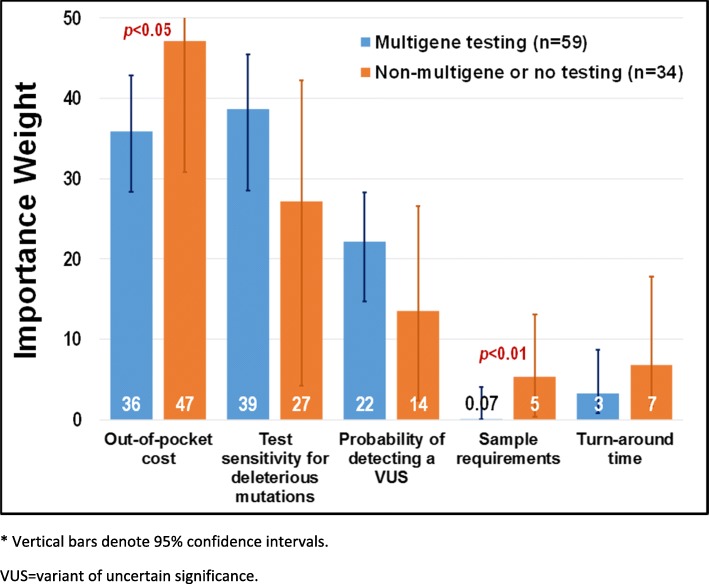
Relative importance weights by patients undergoing multigene testing vs. non-multigene or no testing*. * Vertical bars denote 95% confidence intervals. VUS = variant of uncertain significance

There were several differences noted between the preferences of women who subsequently opted for multi-gene testing (*N* = 55) and those who opted for either single-gene testing or no testing (including those who did not attend a genetics appointment) (*N* = 39). Although not statistically significant at the 5% level, enhanced probability of detection of both deleterious mutations and VUS were more important to women who subsequently chose to undergo multigene testing than to women who chose single gene testing or no testing (Fig. 4). Out-of-pocket cost (*p* < 0.05) and sample requirements (*p* < 0.01) were more important to women who opted for single gene testing or no testing than to women who opted for multi-gene testing (Fig. 3).

## Discussion

Our study is the first to evaluate patient-level preferences for specific attributes of various genetic testing strategies that are available to women with epithelial ovarian cancer. The care of women with advanced EOC is exceedingly complex—decisions regarding anti-cancer treatment, extensive surgeries, and symptom management are made throughout their cancer trajectories. Information regarding patient preferences for various aspects of their care, and in the case of our study, genetic testing options, may assist clinicians in the delivery of ‘patient-centered care’, defined as care that is “respectful of and responsive to individual patient preferences, needs, and values, and ensuring that patient values guide all clinical decisions [14].” Women may also benefit from knowledge of this study demonstrating that others in their situation have similar concerns and preferences regarding cost and/or the ability of a genetic test to detect mutations.

Cancer diagnoses are among the most expensive medical conditions for patients in the United States; some spend more than 20% of their yearly income on cancer-related medical costs [15]. Patients at the highest risk for cancer-related financial toxicity include those with advanced/recurrent cancer, cancers with poor prognoses, those needing chemotherapy and/or radiation, and those with co-existing chronic conditions (such as diabetes or heart-related illnesses) [15]. EOC meets several of these risk factors; it is therefore not surprising that our study identified cost as the most important attribute for women with EOC. While insurance benefits often cover the cost of genetic testing for EOC, concerns over how positive results may affect future insurance benefit coverage and premiums may lead some patients to consider paying for testing out-of-pocket or to decline testing altogether [16].

Tests with higher probability to detect deleterious mutations and a VUS result were preferred by women considering genetic testing for EOC. While we expected patient preferences to align with testing strategies offering higher probability of detection of deleterious mutations, we did not anticipate a preference for strategies identifying incidental, non-actionable VUS. Participants’ preference for VUS results may represent patients’ desire to have as much knowledge as possible despite lack of any clinical guidelines. This is in line with a recent study demonstrating that more than three-fourths of ovarian cancer patients surveyed expressed a desire to obtain their genetic results, even if the information was not currently actionable [17]. The expectation that future research could make VUS results valuable suggests this may be an important topic for genetic counseling.

Overestimation of cancer risk associated with VUS, as well as changes in cancer screening behavior and medical decision making following the finding of a VUS, have been well documented [18, 19]. As genetic testing becomes more widely available, some patients are likely undergoing testing without clear indications. In these situations, identification of VUS becomes increasingly problematic. Clinicians not familiar with VUS implications may recommend unnecessary procedures or even surgeries, putting the patient at risk and inflating health care costs. Recently, a study was published assessing comfort with genetic testing and VUS results across a broad range of specialties [20]. Not only did 75% of physicians make incorrect recommendations for genetic testing, less than 15% were able to correctly interpret test results. Most reported they would not feel comfortable discussing VUS results with their patients [20]. Addressing patient and physician perception of VUS and improved counseling regarding VUS implications is of the utmost importance to avoid unnecessary medical interventions and surgeries for these women.

Based on our data, a woman’s preferences for various attributes of genetic testing *do* influence her decision to undergo testing and, if so, which kind. Not surprisingly, women opting for single gene testing or no testing at all are more concerned about the costs associated with testing. It is unclear why this group also had strong preferences for method of specimen collection; this may be related to perceptions that blood tests may be more costly than a saliva sample. Attributes such as cost, probability of detecting a deleterious mutations, and probability of a VUS result can influence genetic testing decisions and should be incorporated into patient-centered genetic counseling discussions.

Interestingly, nearly 25% of patients in this study did not choose to pursue the counseling appointment. Reasons for this were not evaluated in the current study. Previous publications have identified both clinician and patient-level barriers in the performance of genetic testing. In our study, although all patients were approached by their gynecologic oncologist regarding the importance of genetic testing and potential study enrollment, 4/94 (4.2%) did not have a referral placed. Streamlining the referral process for clinicians may promote referrals for genetic testing. Swanson *et. al* demonstrated the utility of a bundled intervention for clinicians caring for women with EOC. Upon implementation, referrals to genetic counselors increased from 48 to 72% in a 2-year period [21]. In non-academic centers, providers may not be aware of genetic testing services available in their area [22]. The current shortage of genetic counselors is another barrier to testing; however, the supply of counselors is predicted to meet demand between 2024 and 2030 [23]. Finally, previous studies have identified several patient-level barriers to genetic testing, including lack of understanding [24, 25], perceived psychosocial implications, inconvenience of counseling/testing [26], and concerns for potential discrimination. Addressing these concerns, as well as the potential personal and family benefits of testing, is critical if widespread uptake of genetic testing in EOC is to be achieved. Limitations of our study include our choice of only five specific attributes of genetic testing. Other testing characteristics are likely to prove important to patients in their decision to undergo one of a variety of genetic testing strategies now available. In addition, there was a lack of uniformity in the timing of referral to genetic counseling (i.e. front line setting vs recurrent disease) which may affect patient preferences.

## Conclusions

Our study demonstrates that women with EOC have preferences for aspects of genetic testing options which may drive their choice for available testing options. Information regarding variants of uncertain significance is important to patients despite a lack of clinical guidelines. Despite recommendation for genetic testing with a diagnosis of EOC, 25% of patients in our study did not attending counseling appointments. With the information gained from our study, clinicians may engage in more tailored patient-centered conversations to encourage the uptake of genetic counseling and testing. Our study also highlights the need to address VUS prior to testing and following receipt of results. Renewed efforts to counsel patients prior to testing and upon receipt of testing results is of the utmost importance to ensure accurate patient understanding and to avoid unnecessary testing and surgery.

## Additional file

Additional file 1: Subject Education. (DOCX 20 kb)

## Abbreviations

CI: Confidence interval
DCE: Discrete choice experiemtn
EOC: Epithelial ovarian cancer
MEV: money-equivalent values
PARP: poly ADP ribose polymerase
SGO: Society of Gynecologic Oncology
VUS: Variants of uncertain significance
