# Supplementary material for: Preferences of women with epithelial ovarian cancer for aspects of genetic testing
Source: Gynecol Oncol Res Pract. 2019 Jan 22;6:1. doi: 10.1186/s40661-019-0066-8 (PMC6341581; doi:10.1186/s40661-019-0066-8)
Supplement: Supplementary file 1 — Subject Education. (DOCX 20 kb) [file 40661_2019_66_MOESM1_ESM.docx]

**Additional file 1. Subject Education**

Video presentation to subject: The following will be read to the subject in a video presentation with enhancing images.

You are watching this video because you have agreed to participate in a study about your preferences for genetic testing. Approximately 10 out of every 100 women, or 10%, with ovarian cancer have mutations in certain cancer genes, even if they don’t have a family history of cancer. A mutation is a change in a gene. Many genes make up your DNA and are passed down from parents to children. The BRCA genes are the most important genes responsible for ovarian and breast cancers that run in families. Finding out if you have mutations in these genes is important because it may affect your treatment options and your prognosis. Because these mutations may also be present in other family members, this information may also be important to you and your family when considering the need for more effective cancer screening and possible preventive surgeries. Mutations in BRCA genes have also been associated with increased risk of breast, pancreatic, melanoma and prostate cancers. There are other, less common genes that are also important in ovarian cancer. Genetic counselors may talk to you about testing for these other genes as well. If you do have a mutation in any one of these genes, you would be considered a “mutation carrier” for that specific gene.

There are different kinds of genetic testing available. You will soon be asked to evaluate the importance of 5 features in your decision to undergo genetic testing, and, if so, which type of test you prefer. These features are: 1. Out of pocket costs 2. The type of gene test performed 3. The chance of an uncertain result 4. The method of specimen collection 5. Turn around time for results.

**Feature 1: The first feature is out of pocket costs.**

This refers to how much you will need to pay of your own money to have genetic testing done. Some insurance companies may cover all or part of the cost of genetic testing. However, we wish you to focus on how much you would be asked to pay, regardless of what your insurance is willing to pay.

**Feature 2: The second feature is the type of genetic test that you choose.**

Many genes are associated with an increased risk of breast, ovarian, and other cancers. Individual genes can have different types of mutations, some that are known to increase a person’s risk of cancer. For other types of mutations, we don’t yet have enough information to know if having one of these will increase or decrease your risk of cancer.

BRCA-1 and BRCA-2 are the best-known genes that have been linked to an increased risk of ovarian and breast cancer. Forty out of 100 women, or 40%, with BRCA-1 mutations will develop ovarian cancer in their lifetime, while 60 out of 100 women, or 60%, with this mutation will develop breast cancer. The risk is lower for BRCA-2 mutation carriers: approximately 18 of every 100 women, or 18%, will develop ovarian cancer in their lifetime while 47 out of 100, or 47%, with BRCA-2 will develop breast cancer. Finding a mutation in BRCA 1 or 2 may mean that special “targeted” drugs can be used to treat your cancer. These drugs may not be available to patients without these specific mutations.

Mutations in over 10 other genes have also been associated with an increased risk of ovarian cancer. It is unclear what the risk of ovarian cancer is with these less common gene mutations. Mutations in 15 other genes have also been associated with an increased risk of breast cancer. Having a family history of breast or ovarian cancer increases your risk of having a mutation that runs in your family.

There are two types of genetic testing for you to consider today:

BRCA only testing: This means that you are only tested for mutations in BRCA 1 & 2 genes, which are the most common genes associated with hereditary breast and ovarian cancer. It does not tell you anything about the less common genes.

Your other choice is multi-gene testing: This means you are tested for up to 25 different genes, including BRCA1 and 2. Mutations in any one of these genes may influence your risk for several cancers, including breast, ovarian, colon and uterine. The more genes that are tested, the more likely it is that testing will find a mutation.

This question asks in what genes would you be interested in looking for mutations.

1. BRCA only testing
2. Multi- gene testing

**Feature 3. The third feature is the chance of an uncertain variant result.**

Some gene mutations carry a known increased risk of cancer. Other gene mutations, called UNCERTAIN VARIANTS, have no known association with cancer. This is because scientists do not know enough about these variants to know whether they increase your risk for cancer or not. Genetic testing sometimes finds these additional uncertain variants. If you have a genetic uncertain variant, this means there is a genetic abnormality, but doctors don't know what it means. Uncertain variants can be found in any gene, including BRCA 1 and BRCA 2. We don’t know if uncertain variants increase or decrease a women’s risk of breast or ovarian cancer or other types of cancer. If your genetic testing identifies an uncertain variant your doctor will not be able to tell you anything new about your or your family’s cancer risk. However, new information may become available in the next few years about what your variant means.

Different genetic tests have different chances of an uncertain variant result. Some tests have a chance of finding a variant as low as 5 in every 100 women tested or as high as 40 in every 100 women tested (show picture).

This feature represents the chance of finding an uncertain variant when undergoing genetic testing.

5 in 100, or 5%

20 in 100, or 20%

40 in 100, or 50%

1. 5%
2. 20%
3. 40%

**Feature 4: The fourth feature is how the sample is collected.**

Different genetic tests require different methods to obtain your cells for testing.

Saliva means a swab of your mouth or sample of your saliva is used to obtain these cells.

Blood means a blood sample will have to be taken from a vein to obtain cells.

1. Saliva
2. Blood

**Feature 5: The fifth feature is the turn around time for results.**

After collection of your cells, it takes some time to receive the results of your genetic testing. It may take anywhere from 1-4 weeks to know the results of your genetic test.

1. 1 week
2. 2 weeks
3. 4 weeks
